# Effect of Silver Nanoparticle Administration on Productive Performance, Blood Parameters, Antioxidative Status, and Silver Residues in Growing Rabbits under Hot Climate

**DOI:** 10.3390/ani9100845

**Published:** 2019-10-21

**Authors:** Magdy Abdelsalam, Ibrahim Al-Homidan, Tarek Ebeid, Osama Abou-Emera, Mohamed Mostafa, Mohamed Abd El-Razik, Mohamed Shehab-El-Deen, Sherif Abdel Ghani, Moataz Fathi

**Affiliations:** 1 College of Agriculture and Veterinary Medicine, Qassim University, Al-Qassim 51452, Saudi Arabia; mgdy_abdelsalam@yahoo.com (M.A.); homidani@yahoo.com (I.A.-H.); tarkamin@gmail.com (T.E.); osama_emera@yahoo.com (O.A.-E.); mohamedmostaf_2004@yahoo.com (M.M.); mabdelrazik1969@yahoo.com (M.A.E.-R.); m.shehabeldeen@qu.edu.sa (M.S.-E.-D.); sherifbiomy@yahoo.com (S.A.G.); 2 Faculty of Agriculture, Alexandria University, El-Shatby 21545, Alexandria, Egypt; 3 Faculty of Agriculture, Kafrelsheikh University, Kafr El-Sheikh 33516, Egypt; 4 Animal Production Research Institute, Agriculture Research Center, Dokki 12618, Giza, Egypt; 5 Faculty of Agriculture, Ain Shams University, Hadayek Shoubra 11241, Cairo, Egypt; 6 Faculty of Agriculture, Suez Canal University, Ismailia 41522, Egypt

**Keywords:** silver nanoparticles, productive traits, antioxidant, silver residue, Jabali rabbits

## Abstract

**Simple Summary:**

Silver nanoparticles (AgNPs) were used for their antibacterial effects, which increase productive performance and immune response in poultry and rabbits. On the other hand, residues of silver in meat and the internal organs of treated animals may be toxic for human beings. The current results of using two doses of injecting AgNPs revealed that body weight significantly improved in rabbits given a low dose of AgNPs compared with control animals. For consumers’ health concerns, it is of interest to note that the amount of accumulated silver in blood plasma and meat increased dramatically with an increasing dose.

**Abstract:**

The influence of subcutaneous injections of silver nanoparticles (AgNPs) on rabbit performance, hematological and biochemical parameters of blood, antioxidant status, and the residues of silver in meat and blood in two breeds (New Zealand White (NZW) and Jabali) of rabbits growing under high ambient temperature was evaluated. A total of 90 six-week-old rabbits (45 NZW and 45 Jabali) were randomly distributed into three equal treatment groups (control, 0.5 mg, and 1.0 mg AgNPs/kg body weight). The treated rabbits were injected twice a week for four consecutive weeks. The results revealed that AgNPs administration had no significant effect on average daily gain (ADG), feed intake, and feed conversion ratio (FCR). The NZW breed surpassed the Jabali breed in growth performance traits, carcass weight, dressing percentage, and cuts of mid parts and hind cuts. Administration of AgNPs had a significant effect on hematocrit (HCT) and platelet (PLT) values. Rabbits injected with AgNPs at a dose of 0.5 mg showed a lower plasma concentration of total cholesterol and triglycerides than that of control rabbits. The NZW breed had significantly low platelet, total cholesterol, and triglyceride values. Rabbits injected with 0.5 mg/kg BW had the lowest total antioxidant capacity and highest malondialdehyde (MDA) and glutathione peroxidase. The Ag residues were higher in blood than those in meat in treated rabbits. The local breed (Jabali) had significantly lower residues than the imported one (NZW) either in meat or in blood. However, the amount of accumulated silver in blood plasma and meat increased with increasing dose.

## 1. Introduction

The concept of nanotechnology depends on reducing particle size to change the physical and chemical nature of an element. The technology of nanoparticles is widely used in various applications of nutrition, therapy, and medication. Nowadays, nanoparticles are greatly considered in livestock and poultry production because of chemical and physical properties. Silver nanoparticles (AgNPs) are defined as particles sized less than 100 nanometers. Several studies have suggested that feeding of nanoparticles improved the digestive efficiency, immunity, and performance in livestock and poultry. The use of silver nanoparticles as a powerful disinfection agent due to the fact of its antibacterial and antifungal characteristics has led to its recent application in the animal production sector [1,2,3]. Abd Al-Rahman et al. [4] reported that the intraperitonially injection of AgNPs had the ability to increase the immune responses of mice in vivo and in vitro. Rabbits injected intravenously with AgNPs (0.6 mg/kg BW) showed higher seminal reactive oxygen species, less motile sperm, and lower curvilinear velocity and oxygen consumption than control animals. In contrast, libido, serum testosterone, sperm concentration, and semen volume were hardly affected by AgNPs [5]. On the other hand, many reports revealed that AgNPs administration in drinking water did not affect the intestinal microbial profile of broilers [6,7]. Deterioration in growth performance and economic traits were noticed in broilers treated with AgNPs via the drinking water (up to 12 ppm). The application of AgNPs via the drinking water in the concentration of 50 ppm reduced broiler growth, impaired immune functions, and had no antibacterial effect on different intestinal bacterial groups [7]. Moreover, in vivo studies with chicken embryos and quails showed that adding AgNPs via the drinking water did not affect growth and embryonic development [8]. Silver NPs affects N utilization and plasma IgG concentration; however, it does not influence the microbial populations in the digestive tract, energy metabolism, and growth performance of chickens [6].

The introduction of these novel materials into the work environment and consumer products necessitates safety evaluations, as well as clearer understanding of any potential impact on human health [9]. A linear increase of AgNPs retention in meat, liver, and feces with a higher level was detected in male broilers treated with up to 12 ppm nanosilver via drinking water [10]. With respect to AgNPs toxicity, Orlowski et al. [11] reported that silver nanoparticles had a toxic effect and potential to induce inflammatory reactions in mouse monocyte cell line. Likewise, Sardari et al. [12] suggested that feeding AgNPs caused toxicity in rats’ organs. In rats, Abd AL-Rhman et al. [4] elected a dose of 2 mg/kg body weight, while in rabbits, Castellini et. al. [5] elected an injected dose of 0.6 mg/kg BW. However, few studies on the administration of AgNPs on rabbit production and its residues in meat have been conducted. In this context, the present study was performed to answer the following questions. First, can the injection of AgNPs affect the growing rabbit performance? Second, to what extent would the accumulative amount of Ag be found in both the blood plasma and meat of treated animals?

## 2. Materials and Methods

This experiment was carried out at the experimental rabbit farm, College of Agriculture and Veterinary Medicine, Qassim University, during spring season of 2018. The experiment started at 6 weeks of age and lasted 4 weeks. The daily variation of ambient temperature (high and low) during the experimental period was 33 °C ± 0.6 and 18 °C ± 0.3, respectively (mean ± SE). The animal care, handling and sampling procedures were approved by the committee of health research ethics and animal care of Scientific Research Deanship (protocol # 190208), Qassim University, Saudi Arabia.

### 2.1. Husbandry, Diets, and Experimental Design

A total of 90 growing rabbits aged 6 weeks representing two breeds (Jabali, Saudi local breed and New Zealand White) were used in the present study (45 each). The rabbits were randomly assigned to a completely randomized design with a 2 × 3 factorial arrangement of two breeds and three doses of silver nanoparticles (6 sub-groups/15 animals each). The treated groups were subcutaneously injected with 0, 0.5, and 1 mg of nanosilver colloid/kg body weight twice a week for 4 consecutive weeks. All rabbits were kept individually under similar housing and management conditions inside a semi-closed rabbitry in wire mesh cages (50 cm × 40 cm × 40 cm). Each cage unit was equipped with a feeding hopper and drinking nipples providing free access to feed and water. The rabbits were fed a commercial diet containing 18.5% crude protein, 8.0% crude fiber, 3.0% crude fat, and 2250 ME Kcal/kg.

### 2.2. Synthesis of Nanosilver Colloid

The colloid of silver nanoparticles was prepared according to Lee and Meisel [13]. Ten milliliters of 1% sodium tricitrate aqueous solution were added dropwise to 500 mL solution of silver nitrate (2 mM). The mixture was brought to boil under vigorous stirring until the solution turned yellow. The mixture was left to cool down under stirring. A gray suspension of silver nanoparticles was formed. The synthesized particles were subjected to particle size analysis using a laser particle size analyzer (S3500 Bluwave Mictotrac, Brentwood, TN, USA). The mean size of the particles was 43 nm.

### 2.3. Growth Performance and Carcass Characteristics

All rabbits were weighed at the beginning and at the end of the experiment (10 weeks of age). Feed intake was individually recorded throughout the experimental period. The feed conversion ratio (FCR) was calculated as feed consumed divided by weight gain during the experimental period. At the end of the experiment, sixty animals were weighed, fasted for 12 h with free access to clean drinking water, and sacrificed (*n* = 10 animals/each sub-group). Upon bleeding, the rabbits were dissected according to Fathi et al. [14]. After skinning, the carcass was opened and all organs and offal were removed. Hot carcass, skin, liver, heart, kidney, and spleen were excised and weighed. The carcass was divided into three cuts: fore part, mid part, and hind part. All data were expressed as a percentage of live body weight.

### 2.4. Blood Collection, Hematology, and Biochemical Assay

Two blood samples were collected from the carcass of each rabbit into heparinized tubes for determination of hematological parameters and biochemical analysis. The hematological parameters were assessed using an Automatic Fully Digital Hematology Analyzer, BC-3000 Plus (Mindary, Bio-Medical Electronics Co., Ltd, Mahwah, NJ, USA.). These parameters were: total count of red blood cell (RBC), hemoglobin (HGB), hematocrit (HCT), and platelets (PLTs). The other blood samples were centrifuged (1500× *g* for 12 min at 4 °C) and the harvested plasma were stored at −20 °C for further analysis. The concentrations of total protein, albumin, cholesterol, and triglycerides were determined in the plasma using commercial kits (Biomerieux, Craponne, France).

### 2.5. Determination of Antioxidant Status

Total antioxidant capacity (TAC) was determined in mmol/L using a commercial kit (Biodiagnostic^©^ for diagnostic and research reagents, Dokki, Giza, Egypt). This method exploits the ability of antioxidants to reduce hydrogen peroxide (H_2_O_2_). Determination was performed by the reaction of antioxidants in the sample with a defined amount of exogenously provided H_2_O_2_. The antioxidants in the sample eliminate a certain amount of the provided H_2_O_2_. The residual of H_2_O_2_ is quantified colorimetrically by an enzymatic reaction which evolves the conversion of 3,5,dichloro-2-hydroxy benzensulphonate to a colored product. Glutathione peroxidase (GSH-Px) was determined in erythrocytes. The red blood cells were collected from blood samples and washed with saline solution 3 times. Cold deionized water (4 °C) was added to lyse cells. The resulting clarified supernatant was used in GSH-Px assay. Enzyme activity (reduction of organic peroxide) was spectrophotometric monitored by decreasing absorbance at 340 nm. Malondialdehyde (MDA) level was determined from MDA equivalence standard. Samples and standards were first reacted with thiobarbituric acid (TBA) in acidic medium at high temperature (95 °C) for 30 min to form a reactive pink product. The samples and standards were read spectrophotometrically at 534 nm.

### 2.6. Determination of Silver Residues in Blood Plasma and Meat

Samples of 1.5 g dried meat samples (left leg) and 1 mL of plasma were placed in a digestion tube filled with 5 mL HNO_3_ and 2 mL H_2_SO_4_ solution and left to be digested in a hot block digestion apparatus on 60 °C for 30 min. The tubes were then removed and allowed to cool. Ten milliliters of concentrated HNO_3_ were added and returned to the digestion track and slowly heated to 120 °C. The H_2_O_2_ was gradually added to each tube until the solution liquid turned clear. The samples were heated until almost dry and left to cool. The residue in the digestion tube was dissolved in ultra-deionized water up to 100 mL. The amount of silver (Ag) in diluted solution of meat or plasma was determined by ICP-OES (Model iCAP 7400 Duo, Shanghai, China). The figures were adjusted according to the dilution factor.

### 2.7. Statistical Analysis

Data were subjected to a two-way ANOVA using JMP Ver. 11, Cary, NC, USA [15] with an injected dose of silver nanoparticles and breed as fixed effects according to the following model:

Y_ijk_ = µ + D_i_+ B_j_ + (DB)_ij_ + e_ijk_

where:

Y_ijk_ = the observation taken on the kth individual;
µ = overall mean;
D_i_ = the fixed effect of the ith silver nanoparticles injected dose;
B_j_ = the fixed effect of the jth breed;
(DB)_ij_ = interaction between silver nanoparticles injected dose and breed;
e_ijk_ = random error assumed to be independent normally distributed with mean = 0 and variance = σ^2^.

All results are presented as the mean and the variability in data is expressed as pooled standard error of the mean (SEM). The significance of difference among the groups was assessed using Tukey’s test. Statistical significance was considered when *p* < 0.05.

## 3. Results

### 3.1. Growth Performance

The effects of AgNPs administration on body weight (BW), average daily gain (ADG), feed intake (FI), and feed conversion ratio (FCR) are listed in Table 1. Administration of AgNPs had a significant effect on final BW (*p <* 0.02). Rabbits injected with low dose of AgNPs (0.5 mg/kg BW) had, significantly, the heaviest final BW than control animals, showing that those injected with the high dose (1 mg/kg BW) intermediate value. Administration of AgNPs had no significant effect on ADG, FI, and FCR. Regarding the breed effect, the imported breed (New Zealand White) exceeded local breed (Jabali breed) in final BW (*p* < 0.01), ADG (*p* < 0.01), FCR (*p* < 0.01), and FI (*p* < 0.29). The interaction between AgNPs administration and breed had no significant effect on growth performance parameters under high ambient temperature (Table 1).

### 3.2. Carcass Characteristics and Relative Weight Organs

The main effects of AgNPs administration and breed on carcass measurements and internal organs are presented in Table 2. No significant differences were detected for dressing percentage and different parts of the carcass due to the fact of AgNPs administration or breed. A significant difference was found due to the breed effect on carcass weight (*p* < 0.04). The NZW breed had the heaviest carcass weight compared to Jabali rabbits. There were no significant differences for the remaining carcass parts due to the breed effect. It can be noticed that the dose of injected AgNPs had an insignificant effect on the liver, heart, kidney, and spleen percentages (*p* > 0.05). Concerning the breed, Jabali rabbits had a significantly higher (*p* < 0.03) relative percentage of spleen compared to NZW rabbits. A significant interaction between administration and breed was detected in dressing carcass and mid-part percentage, describing a differed behavior for each breed. It is of interest to note that the NZW breed was associated with a higher mid-part for animals treated with AgNPs, while the opposite trend was found in the Jabali breed.

### 3.3. Blood Hematological and Plasma Biochemical Constituents

The influences of AgNPs administration and breed on some hematological parameters and blood plasma constituents are presented in Table 3. Administration of AgNPs had no significant effect on all blood parameters except for HCT and PLT values. Rabbits injected with high dose of AgNPs (1 mg/kg BW) had, significantly (*p* = 0.04), the highest HCT value than control animals, showing those injected with the low dose (0.5 mg/kg BW) an intermediate value. The platelets value was significantly (*p* = 0.02) reduced due to the AgNPs administration in comparison with control rabbits. As shown in Table 3, plasma total protein, albumin, globulin, total cholesterol, and triglycerides concentrations were not significantly affected due to the administration of AgNPs. However, on the basis of breed factor, there were significant differences between breeds for albumin, total cholesterol, and triglycerides concentrations, while no significant difference was detected for total protein and globulin. The NZW breed had a significantly (*p* < 0.05) lower platelets, total cholesterol and triglycerides values compared to the Jabali one. However, the Jabali breed recorded a significantly (*p* < 0.05) lower plasma albumin level compared with the imported breed. The interaction between AgNPs administration and breed had no significant effect for all blood parameters under high ambient temperature.

### 3.4. Antioxidative Properties and Lipid Peroxidation

Data concerning the influence of AgNPs administration and breed and their interaction on blood plasma antioxidative properties are presented in Table 4. It could be observed that there were no significant differences for TAC, GSH-Px, and MDA due to the administration of AgNPs, breed, and their interaction in rabbits growing under high ambient temperature.

### 3.5. Silver Residues in Blood Plasma and Meat

The effect of AgNPs administration, breed, and their interaction on the amount of residues of silver in meat and blood plasma are presented in Table 5. The dose of AgNPs had a highly significant effect on Ag residues in meat (*p* < 0.01). While in blood plasma, an increase (*p* < 0.08) in residues was observed in rabbits injected with a high level compared to those injected with low level. It is clear that the residues in both meat and blood plasma increased as the dose of injected AgNPs increased. On the basis of breed effect, there is a highly significant effect (*p* < 0.01) on Ag residues in meat and blood plasma. The higher amount of Ag was accumulated in the NZW breed compared to the Jabali one. In addition, a significant interaction between the AgNPs administration dose and breed was detected for Ag residues in meat (Table 5). The behavior manner of Ag residues in Jabali meat resulting from the AgNPs injection was dissimilar to that of NZW breed.

## 4. Discussion

### 4.1. Growth Performance

As shown in Table 1, administration of AgNPs had a significant effect on final BW under high ambient temperature (*p* < 0.02). These results are in agreement with Hang and Tra [16] who found that BW of rabbits given AgNPs in drinking water was higher than that of the control group. They added that there were no differences in FI among both groups. In broiler chickens, Andi et al. [17] and Hassan [18] found that broilers treated with AgNPs showed heavier BW and high BWG than those of the control. The authors attributed this increase to the biological effect of AgNPs on harmful bacteria in intestine which resulted in improved growth as the absorption of nutrients was increased [19]. In addition, the AgNPs growth stimulatory effect might be attributed to the stimulation of digestive enzymatic activity which resulted in improving the absorbance of nutrients [20]. On the contrary, several previous studies did not find an improvement in growth performance in broilers fed a diet supplemented with AgNPs [10,21,22]. Similar findings were detected in the present study. Administration of AgNPs had no significant effect on BWG, FI, and FCR (Table 1). These contradictory findings might be attributed to the variations in the AgNPs size, dose, exposure time, and preparation method.

### 4.2. Carcass Characteristics

It can be noticed that the dose of AgNPs had no significant effect on carcass weight, dressing percentage, and different parts of carcass, such as the liver, heart, kidney, and spleen percentages (*p* > 0.05). Similarly, in rats, Kim et al. [23] reported that oral administration of AgNPs had an insignificant effect on the relative weights of spleen, liver, heart, and kidney. On the other hand, Abd [24] found an increase in the spleen percentage of rabbits injected with AgNPs compare to the control rabbit. In broiler chickens, Hassan [18] found that AgNPs supplementation in drinking water or diet had a significant effect on dressing %, liver %, and heart %. The results of the present study indicated that breed had a significant effect on spleen percentage. The Jabali breed had higher relative percentage of spleen and this may be attributed to genotypic differences (Table 2). Pla et al. [25] reported that the breed of rabbit had a highly significant effect on dressing percentage, liver weight, and hind part weight. Wang et al. [26] found that breed had a significant effect on head, skin, liver, and kidney percentages. Metzger et al. [27] pointed out that liver, heart, and kidney are early-maturing organs and rabbits with high gain have an earlier development. On the other hand, Ghosh and Mandal [28] found that there was no significant difference in organ weights among studied breeds.

### 4.3. Blood Hematological and Plasma Biochemical Constituents

Administration of AgNPs had no significant effect in all blood parameters except for HCT and PLT values (Table 3). Similarly, Syrvatka et al. [29] reported that all blood parameters studied were not significantly affected by AgNPs treatments in NZW female rabbits. Raheem [30] found that there was an increase in RBCs, PLTs, and HCT values in rabbits immunized with AgNPs compared to the control rabbits. Sarhan and Hussein [31] found that AgNPs injected intravenously resulted in changes in the values of PLTs and RBCs, and they attributed the change in RBCs to increased immunogenic response or disturbances in signaling pathways and maturation of cells. Atmaca et al. [32] reported that the decrease in the PLT counts was due to the harmful effects on hematopoietic organs. Data of the present study illustrated that administration of AgNPs in a high dose significantly (*p* = 0.04) increased HCT values compared to the control animals. An insignificant effect on HGB and RBCs due to the dose injection was found, indicating that this treatment had no deleterious effect on growing rabbits under high ambient temperature. Plasma total protein, albumin, globulin, total cholesterol, and triglycerides concentrations were not significantly affected due to the administration of AgNPs. On the contrary, Saleh and El-Magd [20] reported that plasma triglycerides and total cholesterol were significantly lower in the broiler chicks fed a diet supplemented with AgNPs, while plasma total protein were not influenced compared to those in the control group. Ahmadi [33] illustrated that broiler chicks fed AgNPs exhibited significant changes in total protein, albumin, and gamma globulin. Regarding the breed effect, there were significant differences in albumin, total cholesterol, and triglycerides concentrations. No significant difference was detected in total protein and globulin. The NZW breed had a significantly (*p* < 0.05) lower platelet, total cholesterol, and triglycerides values compared with the Jabali breed. However, the Jabali breed recorded a significantly (*p* < 0.05) lower plasma albumin level compared with the imported breed. Contrary to the present results, Fathi et al. [14] reported that the local breed (Jabali) had higher HGB, RBC, and HCT figures than the imported breed (Spanish V-line). El-Sheikh et al. [34] reported that native breeds (Black Baladi and Gabali) had higher blood parameters values than imported breeds (NZW and V-Line).

### 4.4. Antioxidative Properties and Lipid Peroxidation

The data in Table 4 prove that AgNPs administration, breed, and the interaction had no significant effect on the total antioxidant capacity, GSH-Px activity, and MDA concentration in the rabbits growing under high ambient temperature. These results indicated that the administration of 0.5 or 1.0 mg AgNPs/kg BW did not induce the oxidative stress in rabbits under heat stress conditions. However, administration of higher doses of AgNPs (5 mg/kg/day) induced hepatic damage and oxidative stress illustrated by a significant decrease in total antioxidant capacity level and the activities of GSH-Px and superoxide dismutase in rats [35]. The decreased levels of GSH-Px due to the injection of AgNPs might be attributed to the high affinity of nanoparticles for thiol groups leading to a decrease in glutathione content due to the free radical scavenging [36]. They attributed this decrease in GSH-Px activity to the increasing use of GSH-Px to downplay the effect of free radicals after exposure to AgNPs. In Table 4, administration of 0.5 or 1.0 mg AgNPs/kg BW had no significant effect on MDA concentration in rabbits. In contrast, Ognik et al. [3] noticed that there was an increase in the plasma content of MDA and a reduction in level of GSH-Px as a result of AgNPs treatment in chickens. They reported that AgNPs treatment causes an oxidative stress leading to reduce in activity of antioxidant enzymes in blood plasma and damage in liver cells. It could be documented that low doses of AgNPs might have no detrimental effects on antioxidant properties in growing rabbits under high ambient temperature.

### 4.5. Silver Residues in Blood Plasma and Meat

The results of the present study revealed that the dose of injected AgNPs had a highly significant effect on Ag residues in meat (*p* < 0.01) in a dose-dependent manner (Table 5). A similar trend was found in blood plasma residues but in an insignificant manner due to the high SEM. These results are in harmony with several previous studies [10,20], which stated that the dietary AgNPs supplementation increased the concentration of Ag in the muscles of broiler chickens. As shown in Table 5, the highest residues were observed in blood rather than in meat which may be due to the fact that AgNPs are able to enter the blood circulation and then distribute to the organs and muscles. Kim et al. [23] and Raheem [30] reported that blood is the center distribution to organs. Fondevila [37] found that Ag retention in liver was higher than that in muscular tissue in broiler chickens. Borel and Sabliov [38] reported that nanomaterials sized 10–100 nm remain for a longer time in the blood circulation before its transfer to the organs and nanoparticles can retain and accumulate in the body rather than excreta. Distribution of nanoparticles could lead to increased interactions with proteins, leading to the biological and physiological changes of cells [39]. Moreover, Kulak et al. [40] reported that AgNPs residues increased in the intestine as their particles size increased (25 or 40 nm) in chicken. They concluded that the large size of nanoparticles had low penetration into bloodstreams. Wang et al. [41] suggested that the liver is considered the major distribution organ followed by the spleen. Nabinejad [2] stated that the muscles and organs of the poultry may transfer AgNPs to consumers which may cause side effects. In terms of dose by breed interaction, it could be concluded that the Jabali breed had a better clearance and lower accumulation of Ag particles in meat compared to the NZW animals. However, the implications of nanosilver in the environment may provide an important context. The environmental risks from silver itself may be mitigated by a tendency of the silver ion to form strong complexes that are apparently of very low bioavailability and toxicity [42]. Regarding breed effect, it could be observed that the local breed (Jabali) exhibited lower residues in both meat and blood plasma compared to the NZW breed. Also, a significant interaction between the AgNPs level and breed was detected in silver residues in meat. However, further studies are needed to evaluate various genotypes of rabbits treated with AgNPs in different administration methods.

In conclusion, injecting rabbits with AgNPs had no significant effect on the average daily gain and feed conversion ratio compared to the control animals without penalizing carcass quality, biochemical blood parameters, and antioxidant profile. From the consumers’ health concern, it is of interest to note that the amount of accumulated silver in blood plasma and meat increased dramatically with an increasing dose. The Jabali breed showed a higher efficiency in eliminating silver residues from blood and meat.

## Figures and Tables

**Table 1:** Effect of the silver nanoparticles (AgNPs) injected dose and breed on body weight, ADG, FI, and FCR in two breeds of rabbits.

Effect		Initial Body Weight (g)	Final Body Weight (g)	ADG (g)	Feed Intake (g)	FCR (g/g)
AgNPs (D)	Control	958.2	1704.4 ^b^	26.6	1620.7	2.39
	0.5 mg/kg	1020.7	1893.9 ^a^	28.8	1748.0	2.20
	1.0 mg/kg	995.3	1724.8 ^b^	24.4	1593.9	2.41
Breed (B)	NZW	984.6	1861.0 ^a^	30.2 ^a^	1697.1	2.08 ^b^
	Jabali	1020.1	1687.7 ^b^	22.9 ^b^	1561.2	2.94 ^a^
SEM		31.2	34.0	1.08	59.5	0.11
*p*-Value	D	0.51	0.02	0.25	0.23	0.66
	B	0.69	0.01	<0.01	0.29	<0.01
	D × B	0.52	0.32	0.35	0.28	0.86

ADG = average daily gain; FCR = feed conversion ratio; ^a,b^ Values within a column and effect with different superscripts differ significantly.

**Table 2:** The effect of the AgNPs injected dose and breed on carcass traits and internal organs as a percentage of body weight.

Effect		Body Weight (g)	Dressing Out (%)	Fore Part (%)	Mid-Part (%)	Hind Part (%)	Liver (%)	Heart (%)	Kidney (%)	Spleen (%)
AgNPs (D)	Control	1823.05	49.29	15.07	14.28	20.39	3.32	0.28	0.42	0.11
	0.5 mg/kg	1835.71	48.85	14.90	13.45	20.50	3.49	0.31	0.41	0.10
	1.0 mg/kg	1813.18	48.59	15.26	13.45	19.89	3.25	0.29	0.38	0.09
Breed (B)	NZW	1893.35 a	49.06	14.98	13.87	20.43	3.40	0.30	0.39	0.09 ^b^
	Jabali	1697.29 b	48.44	15.33	13.14	20.08	3.30	0.29	0.42	0.12 ^a^
SEM		52.88	0.66	0.22	0.36	0.26	0.09	0.01	0.01	0.01
*p*-Value	D	0.58	0.66	0.83	0.29	0.40	0.63	0.44	0.49	0.59
	B	0.04	0.60	0.84	0.62	0.43	0.97	0.74	0.06	0.03
	D × B	0.05	0.07	0.07	0.04	0.35	0.15	0.62	0.09	0.85

^a b^ Values within a column and effect with different superscripts differ significantly.

**Table 3:** Effect of the AgNPs injected dose and breed on the blood hematology and blood biochemical parameters in rabbits.

Effect		HGB (gm/dL)	RBC (10^6^/µL)	HCT (%)	PLT (10^6^/mL)	Total Protein (g/dL)	Albumin (g/dL)	Globulin (g/dL)	Cholesterol mg/dL	Triglycerides mg/dL
AgNPs (D)	Control	12.22	5.38	34.33 ^b^	298.89 ^a^	6.38	3.37	3.01	94.0	137.3
	0.5 mg/kg	13.06	5.67	36.54 ^ab^	237.69 ^b^	6.44	3.47	2.97	87.2	130.2
	1.0 mg/kg	13.33	5.76	37.63 ^a^	221.60 ^b^	6.21	3.51	2.69	110.1	122.1
Breed (B)	NZW	13.01	5.64	36.67	225.78 ^b^	6.39	3.51 ^a^	2.88	90.0 ^b^	120.7 ^b^
	Jabali	12.89	5.64	36.01	286.23 ^a^	6.24	3.37 ^b^	2.87	110.7 ^a^	144.4 ^a^
SEM		0.25	0.08	0.51	11.11	0.11	0.05	0.10	6.3	5.5
*p*-Value	D	0.67	0.56	0.04	0.02	0.68	0.25	0.61	0.10	0.70
	B	0.96	0.95	0.86	0.04	0.38	0.05	0.92	0.05	0.05
	D×B	0.25	0.44	0.18	0.48	0.75	0.38	0.80	0.19	0.59

^a,b^ Values within a column and the effect, and different superscripts differ significantly. HGB = hemoglobin; RBCs = red blood cells; HCT = hematocrit; PLTs = platelets.

**Table 4:** Effect of the AgNPs injected dose and breed on antioxidant traits in rabbits.

Effect		TAC (m mol/L)	GSH-Px (U/mL)	MDA (n mol/mL)
AgNPs (D)	Control	0.94	35.66	3.76
	0.5 mg /kg	0.88	35.83	3.86
	1.0 mg /kg	0.93	33.19	3.76
Breed (B)	NZW	0.92	33.59	3.61
	Jabali	0.91	37.41	4.17
SEM		0.02	2.82	0.21
*p*-Value	D	0.36	0.78	0.99
	B	0.97	0.38	0.31
	D × B	0.86	0.61	0.85

TAC = total antioxidant capacity; GSH-Px = glutathione peroxidase; MDA = malondialdehyde.

**Table 5:** Effect of the AgNPs injected dose and breed on residues of silver in meat and blood plasma in rabbits.

Effect		Meat (µg kg^−1^)	Blood (µg mL^−1^)
AgNPs (D)	Control	Non-detected	Non-detected
	0.5 mg/kg	7.73 ^b^	74.99
	1.0 mg/kg	47.97 ^a^	118.13
Breed (B)	NZW	34.78 ^a^	101.49 ^a^
	Jabali	10.69 ^b^	20.01 ^b^
SEM		2.25	15.71
*p*-Value	D	<0.01	0.08
	B	0.01	0.01
	D × B	0.01	0.13

^a,b^ Values within a column and effect, and different superscripts differ significantly; non-detected = 0 value.
